# Personalized Medicine Approach in a DCM Patient with *LMNA* Mutation Reveals Dysregulation of mTOR Signaling

**DOI:** 10.3390/jpm12071149

**Published:** 2022-07-15

**Authors:** Balram Neupane, Kabita Pradhan, Audrey Magdalena Ortega-Ramirez, Parwez Aidery, Vytautas Kucikas, Matthias Marks, Marc A. M. J. van Zandvoort, Karin Klingel, Klaus K. Witte, Stefan Gründer, Nikolaus Marx, Michael Gramlich

**Affiliations:** 1 Department of Cardiology, Angiology and Critical Care (Medical Clinic 1), University Hospital RWTH Aachen, 52074 Aachen, Germany; bneupane@ukaachen.de (B.N.); kpradhan@ukaachen.de (K.P.); kwitte@ukaachen.de (K.K.W.); nmarx@ukaachen.de (N.M.); 2 Institute of Physiology, RWTH Aachen University, 52074 Aachen, Germany; aortegaramir@ukaachen.de (A.M.O.-R.); sgruender@ukaachen.de (S.G.); 3 Department of Cardiology and Angiology, University Hospital of Tübingen, 72076 Tübingen, Germany; parwez.aidery@med.uni-tuebingen.de; 4 Institute for Molecular Cardiovascular Research (IMCAR), RWTH Aachen University, 52074 Aachen, Germany; vkucikas@ukaachen.de (V.K.); mamj.vanzandvoort@maastrichtuniversity.nl (M.A.M.J.v.Z.); 5 Clinic for Neurology, Section Neurobiological Research, University Hospital RWTH Aachen, 52074 Aachen, Germany; mmarks@ukaachen.de; 6 Interdisciplinary Centre for Clinical Research (IZKF) Aachen, Transgenic Service, University Hospital RWTH Aachen, 52074 Aachen, Germany; 7 Cardiovascular Research Institute Maastricht (CARIM), Department of Molecular Cell Biology, Maastricht University, 6229 ER Maastricht, The Netherlands; 8 Cardiopathology, Institute for Pathology, University Hospital of Tübingen, 72076 Tübingen, Germany; karin.klingel@med.uni-tuebingen.de

**Keywords:** familial DCM, lamin mutation, laminopathy, mTOR inhibitor, individualized therapy

## Abstract

Background: Mutations in the *Lamin A/C* (*LMNA*) gene are responsible for about 6% of all familial dilated cardiomyopathy (DCM) cases which tend to present at a young age and follow a fulminant course. Methods: We report a 47-year-old DCM patient with severely impaired left ventricular ejection fraction and NYHA functional class IV despite optimal heart failure treatment. Whole-exome sequencing revealed an LMNA E161K missense mutation as the pathogenetic cause for DCM in this patient. We generated a patient-specific *LMNA*-knock in (*LMNA*-KI) in vitro model using mES cells. Results: Beta adrenergic stimulation of cardiomyocytes derived from *LMNA*-KI mES cells resulted in augmented mTOR signaling and increased dysregulation of action potentials, which could be effectively prevented by the mTOR-inhibitor rapamycin. A cardiac biopsy confirmed strong activation of the mTOR-signaling pathway in the patient. An off-label treatment with oral rapamycin was initiated and resulted in an improvement in left ventricular ejection fraction (27.8% to 44.5%), NT-BNP (8120 ng/L to 2210 ng/L) and NYHA functional class. Conclusion: We have successfully generated the first in vitro model to recapitulate a patient-specific LMNA E161K mutation which leads to a severe form of DCM. The model may serve as a template for individualized and specific treatment of heart failure.

## 1. Highlights

Elevated mTOR signaling was evident in a DCM patient with an LMNA E161K mutation as well as in cardiomyocytes derived from mouse embryonic stem (mES) cells with the patient-specific *LMNA* mutation.

LMNA E161K mutation resulted in impaired electrophysiological properties and disorganisation of nuclear lamin.

The mTOR-inhibitor rapamycin lead to a rapid reversal of the phenotype in vitro and also improved the clinical status of the patient with DCM and an LMNA E161K mutation.

## 2. Introduction

Dilated cardiomyopathy (DCM) is characterized by left ventricular dilatation and impaired contractility, ventricular and atrial arrhythmias, and a shortened life expectancy due to sudden death or progressive heart failure [1]. Around 20–30% of cases of DCM are thought to be attributable to familial forms. The molecular mechanisms and their genetic origins driving the DCM pathology are complex, due to the possible involvement of more than 40 genes encoding proteins of diverse function and localization such as cytoskeleton, sarcomere, nuclear membrane proteins, and transmembrane phosphoproteins [2,3]. One well-recognized genetic abnormality is that of lamin (LMNA), a series of proteins that provide structural support to the nucleus and assist in chromatin organization, gene regulation, DNA replication, and RNA splicing [4,5,6]. When compared with other known genetically-driven cardiomyopathies, those associated with *LMNA* mutations are associated with a poor clinical outcome [7]. Although a comprehensive analysis of familial history and a genetic assessment are important in assessing the prognosis of the proband and other family members [8] despite considerable enthusiasm for what might be possible, currently this information has no bearing on treatment [9].

Genetic defects result in a wide variety of alterations of proteins which are thought to either lead directly to, or contribute to susceptibility to, pathological cardiac remodeling. However, there is a significant knowledge gap in the nature of the molecular abnormalities and how these translate to the physiological or pathological remodeling process. As a result, there is as yet, little practical utility of genetic approaches in the treatment of DCM [10]. Specifically, in vivo research on *LMNA* mutations is further hindered by the lack of an optimal animal model for *LMNA* cardiomyopathies and also that the clinical phenotypes resulting from *LMNA* mutations, also known as laminopathies, are highly variable due to the involvement of other organ systems and accelerated aging syndrome (progeria) [7]. A hierarchical cluster analysis of 91 mutations in the *LMNA* gene revealed that the organ involvement, type, and severity depend upon the region of mutation in the gene [11].

Here, we report a case of a 47-year-old male patient with severe heart failure due to reduced ejection fraction (HFrEF). He had marked left ventricular (LV) dilatation and systolic dysfunction, New York Heart Association functional class IV symptoms, and persistently raised natriuretic peptide despite optimally tolerated medical therapy.

Whole-exome sequencing revealed a missense mutation (LMNA E161K) as the molecular cause for DCM in the family. This particular mutation is associated with a fulminant course [12] fitting with the clinical situation. This mutation has been associated with abnormal nuclear membrane assembly, genomic clustering of mutated genes, significant changes in nuclear localization and redirected internal areas of the nucleus [13].

Given the severe phenotype associated with this mutation, we aimed to create a patient-specific knock in (KI)-in vitro model to serve as a unique model with which to explore pathological mechanisms and to test individualized treatment approaches.

## 3. Methods

### 3.1. Cell Culture

NIH/3T3 cells (ATCC, CRL-1658^TM^) were used for single guide RNA (sgRNA) optimization. Mouse embryonic fibroblasts (MEFs) (ATCC, SCRC-1008^TM^) were used as feeders for mouse embryonic stem (mES) cells. R1/E mES cells (ATCC, SCRC-1036^TM^) were used as wild type (WT) control and for the generation of a patient-specific knock in the *LMNA* mutation cell line. All cells were handled and cultured according to the protocols provided by ATCC. MEFs were mitotically arrested by irradiation.

### 3.2. CRISPR-Cas9 Mediated LMNA Knock in Mutation in WT mES Cells

sgRNA design: sgRNAs were designed (http://crispor.tefor.net/, accessed on 22 June 2022) and their efficacies were validated in NIH/3T3 cells. sgRNA was synthesized in vitro (IDT DNA, Leuven, Belgium) and used for electroporation into mES cells.

Electroporation protocol: NIH/3T3 or WT mES cells were mixed with CRISPR-Cas9 components (IDT-DNA, Leuven, Belgium) and electroporated via Amaxa^TM^ 4D-Nucleofector^TM^ (Lonza, Cologne, Germany) using the manufacturer’s guide. The extended electroporation protocol is included in the Supplementary Methods.

Surveyor nuclease assay: sgRNAs were first validated in NIH/3T3 cells via hetero-duplexes formed by CRISPR-Cas9 mediated gene editing, reaction buffer, and T7E1 endonuclease (NEB, Frankfurt, Germany). sgRNA with the highest efficacy was used for WT mES cell gene edit.

After electroporation, mES cells were seeded, single colonies were picked, expanded, and further assessed via PCR and restriction digestion. This was done by the *Hind* III restriction enzyme (NEB, Frankfurt, Germany). Positive clones were sent for Sanger sequencing (LGC genomics, Berlin, Germany).

### 3.3. Cardiomyocyte Differentiation of mES Cells

WT or *LMNA*-KI mES cells were differentiated into cardiomyocytes by the previously described hanging drop method [14,15]. Briefly, mES cells were grown in mES cell medium on MEF feeder cells. After two passages, cells were trypsinized, centrifuged, re-suspended in differentiation medium, plated for 30 min to get rid of feeders, plated on the lid of Petri dishes, inverted, and cultured for 3 days. After 3 days, the embryoid bodies were transferred to gelatin-coated 6-well-plates and cultured for the next 12 days. The contracting cells were visible from day 4 onwards which peaked on days 10–12.

### 3.4. Real-Time PCR, Western Blot and Immunohistochemistry

Evaluation of mRNA expression by real-time PCR and detection of protein expressions by Western blot or immunohistochemistry were performed by standard methods. Extended protocols are included in the Supplementary Methods S1.

### 3.5. Electrophysiological Recordings

Standard whole-cell patch-clamp technique was used under the current-clamp configuration, to record repetitive action potentials during spontaneous contractions of WT cardiomyocytes (WT.CMs) or cardiomyocytes derived from *LMNA*-KI mES cell (*LMNA*-KI.CMs) at day 12. Before the patch clamp experiments, the cardiomyocytes were grown on coverslips coated with 0.2% gelatin. The coverslips were then mounted on the stage of an inverted phase-contrast microscope (Olympus IX71, Olympus Corporation, Tokyo, Japan) and prepared for patch clamp. Cells were perfused with Iscove’s 20% FBS medium using an 8-channel gravity-automated perfusion system, cFlow (Cell MicroControls, Norfolk, VA, USA). A random subsample of cells was separated and incubated for 24 h with isoprenaline (2 μM final concentration) or with isoprenaline plus rapamycin (500 nM final concentration) before the measurements. Micropipettes (4–6 MΩ) were prepared from borosilicate glass capillaries with a micropipette puller (DMZ-Universal Electrode Puller; Zeitz Instruments, Martinsried, Germany) and filled with an intracellular solution containing (in mM): 120 KCl, 2 MgCl_2_, 10 HEPES, 10 EGTA and 3 Mg-ATP (pH 7.2). Action potentials were directly recorded from spontaneously firing cardiomyocytes by current clamp without electrical stimulus, using an Axon-200B amplifier (Molecular Devices; San Jose, CA, USA) and an Axon Digidata 1440 A acquisition system controlled by Clampex 10.0 (pClamp 10.0, Molecular Devices, Sunnyvale, CA, USA). Signals were recorded with a 5 kHz sampling rate, and digital data were stored in a compatible PC for off-line analysis using Clampfit 10.0 (pClamp 10.0, Molecular Devices, Sunnyvale, CA, USA).

### 3.6. Video Imaging

The cardiomyocytes differentiated from WT and *LMNA*-KI mutant mES cells were seeded in 6-well-plates. Videos of each well for the contracting cardiomyocytes were taken after 12 days. Each beating was recorded for 1 min. Videos were captured with a camera (The Imaging Source DFK 72AUC02) mounted onto a Leica DM IL LED microscope. The analysis of the video recordings took place in a blinded manner to remove any risk of bias in the assessment.

### 3.7. Patient Study

The patient gave his informed consent to participate in this study. The study was approved by the local ethics committee of the University of Tübingen (Approval code: 270/2011 B01). The investigation conformed to the principles outlined in the Declaration of Helsinki.

### 3.8. Statistical Analysis

All values are presented as mean ± SEM. Unpaired t-test or one-way ANOVA with Tukey’s post hoc analysis was used. A *p*-value of <0.05 was considered to be significant. All data were analysed using GraphPad Prism 9.0 (GraphPad Software, Inc., San Diego, CA, USA).

## 4. Results

### 4.1. Identification of a DCM Patient with an LMNA Mutation

A 47-year-old male patient was referred to our hospital for further evaluation with a long-standing diagnosis of DCM since 2011. Despite optimal guideline-directed heart failure therapy (ramipril 5 mg od, bisoprolol 5 mg od, spironolactone 25 mg od, and torasemide 20 mg od) and the implantation of a cardiac resynchronisation (CRT-D) device in 2015, the patient had severe symptoms, persistent left ventricular dilation (LV end diastolic diameter 58 mm) and reduced left ventricular ejection fraction of 27%. Immunoadsorption of beta-1 adrenergic autoantibodies also failed to improve the clinical status which was further exacerbated by co-morbidities, including myelodysplastic syndrome and recurrent pulmonary embolisms. In addition, coronary angiography excluded the presence of coronary artery disease.

**Figure 1:**
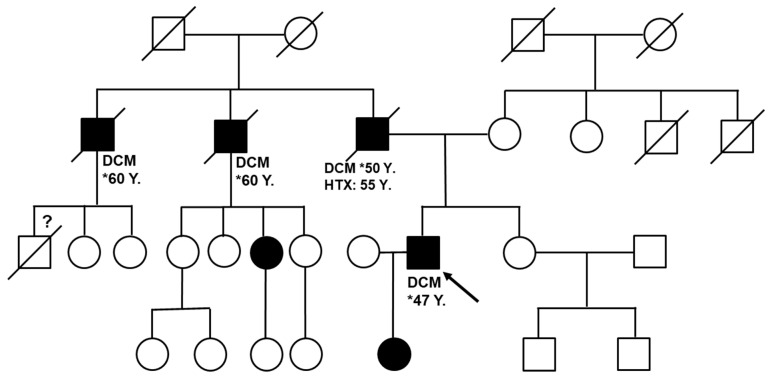
Patient pedigree. The proband is a 47-year-old male (depicted by an arrow). Square boxes denote males while the circles denote females. Squares or circles filled with dark areas denote individuals with the *LMNA* mutation (NM_170707.3: c. 481 G > A: p. E161K). Crossed squares or circles denote that individuals have already passed away. Y = years, DCM = dilated cardiomyopathy, HTX = heart transplantation, * year of DCM diagnosis.

Since the patient had a family history of DCM with his father and his daughter also affected, whole-exome sequencing was performed, which revealed a missense mutation in the *Lamin A/C* (*LMNA*) gene (NM_170707.3: c481 G > A: p. E161K) as the molecular cause for DCM in the family (Figure 1). To further understand the mechanisms of the disease and to establish an individualized treatment approach, we decided to analyse this specific patient mutation in vitro using an mES cell line.

### 4.2. Generation of CRISPR-Cas9 Mediated Patient Specific Knock-In Mouse Embryonic Stem Cells and Cardiomyocyte Differentiation

**Figure 2:**
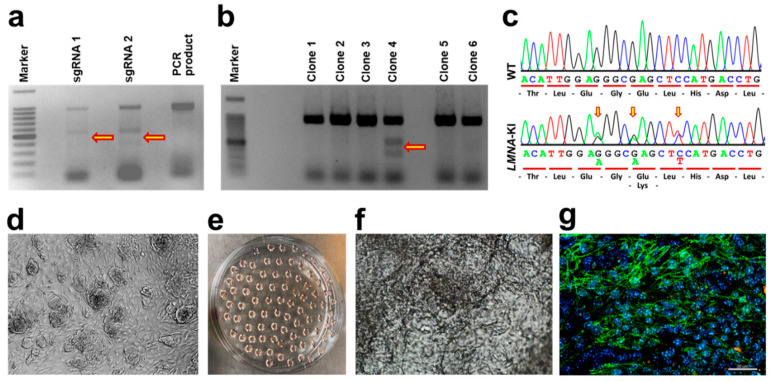
CRISPR-Cas9 mediated patient-specific knock in mES cell line generation and differentiation into cardiomyocytes. (**a**) Surveyor nuclease assay to validate two different sgRNAs (1 and 2) for use in mES cells. Arrows indicate the successful cleavage of PCR products by sgRNAs. (**b**) *Hind* III restriction digest of PCR product of the target region to identify the insertion of donor DNA and presence of a specific mutation in single mES cell clones. Arrow indicates successful cleavage of PCR product. (**c**) Confirmation of positive KI clone via Sanger sequencing. Arrows indicate the insertion of the desired mutation in the mES cell genome. (**d**) WT mES cells are grown on MEF feeder cells. (**e**) Hanging drop culture to initiate cardiomyocyte differentiation from mES cells. (**f**) Differentiated cardiomyocyte from WT mES cells as seen on Day 12 in brightfield microscope. (**g**) Actinin (green) and DAPI (blue) staining in WT cardiomyocytes as seen on Day 12. sgRNA = single guide RNA. Scale bar: 10 μm.

Efficacies of different sgRNAs were validated in NIH/3T3 cells via the Surveyor nuclease assay (Figure 2a). The best sgRNA (Figure 2a, 3rd lane, sgRNA2) was selected for electroporation into mES cells. Forty-eight hours after electroporation, single cell colonies were picked, passaged, and analysed via PCR and subsequent restriction digest (Figure 2b). One positive clone (Figure 2b, 5th lane) was further confirmed via Sanger sequencing (Figure 2c). No off-target effects were detected. Figure 2d and e represent WT mES cells colonies and the hanging drop method respectively. The WT mES cells from now on would be referred to as WT and the patient-specific mutation will be referred to as *LMNA*-KI.

After 12 days of cardiomyocyte differentiation, WT and *LMNA*-KI cardiomyocytes (WT.CM and *LMNA*-KI.CM) were analysed for differentiation markers (Supplementary Table S1, Supplementary Result S1, and Supplementary Figure S1). WT.CMs are depicted as seen by brightfield microscopy (Figure 2f) and by fluorescence microscopy after cardiomyocyte specific actinin antibody staining (Figure 2g).

### 4.3. LMNA-KI Cardiomyocytes Show Increased mTOR Signaling

**Figure 3:**
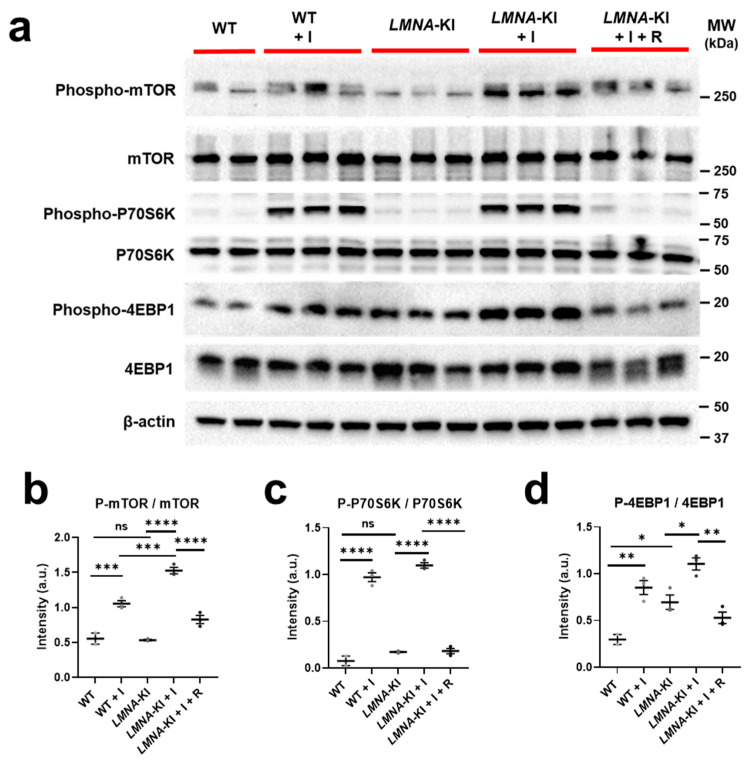
Expression of proteins of the mTOR signaling pathway with and without isoprenaline stimulation. WT and *LMNA*-KI mES cells were differentiated into cardiomyocytes and stimulated on day 12 with either isoprenaline (I) or isoprenaline together with rapamycin (R). (**a**) Western blot images of total and phosphorylated protein levels of mTOR, P70S6K, and 4EBP1. β-actin is used as a loading control. (**b**–**d**) Densitometric quantification of phosphorylated protein relative to total protein, measured by ImageJ program. One-way ANOVA and Tukey’s post hoc analysis were used. Data are shown as mean ± SEM (*n* = 2–3). **** *p* < 0.0001, *** *p* < 0.001, ** *p* < 0.01, * *p* < 0.05. I = isoprenaline, R = rapamycin.

WT.CMs and *LMNA*-KI.CMs were subjected to stress stimulation with the non-selective β-adrenoceptor agonist isoprenaline for 6 h. Moreover, *LMNA*-KI.CMs exposed to isoprenaline were also treated with mTOR (mammalian target of rapamycin) inhibitor rapamycin. Isoprenaline pre-treatment significantly increased the phosphorylation of mTOR (0.9 fold in WT.CM, *p* < 0.001 and 1.9 fold in *LMNA*-KI.CM, *p* < 0.0001), P70S6K (ribosomal protein S6 kinase beta-1) (6.8 and 5.8 fold in WT.CM and *LMNA*-KI.CM respectively, *p* < 0.0001) and 4EBP1 (4E-binding protein 1) (1.2 fold in WT.CM, *p* < 0.01 and 0.6 fold in *LMNA*-KI.CM, *p* < 0.05) (Figure 3a–d). In addition, phosphorylation of mTOR after isoprenaline treatment was significantly higher in *LMNA*-KI.CMs than in WT.CMs (0.5 fold, *p* < 0.001) (Figure 3a,b). Treatment with rapamycin was able to prevent the phosphorylation of mTOR (0.5 fold decrease, *p* < 0.0001), P70S6k (0.8 fold decrease, *p* < 0.0001), and 4EBP1 (0.5 fold decrease, *p* < 0.01) in *LMNA*-KI.CMs (Figure 3a–d). 

### 4.4. LMNA-KI Cardiomyocytes Are Associated with Increased Nuclear Blebbing and Disorganized Lamin Distribution

**Figure 4:**
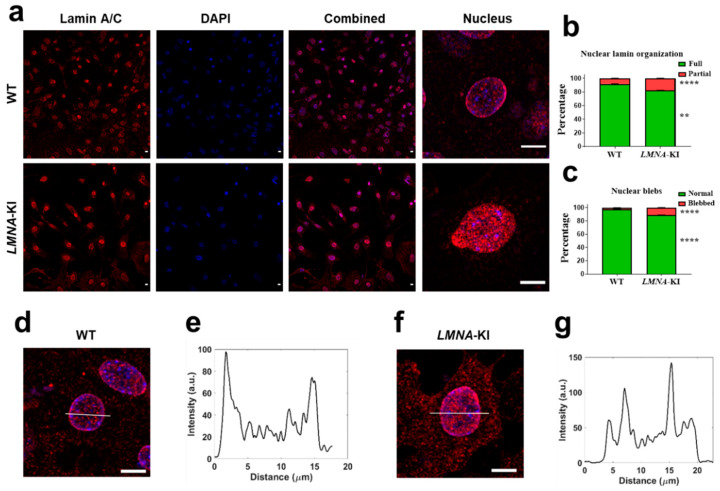
Lamin staining of cardiomyocytes differentiated from WT and *LMNA*-KI mES cells. WT mES cells and *LMNA*-KI mES cells were differentiated into cardiomyocytes and on day 12 stained with lamin antibody and DAPI (panels **a**,**d**,**f**). Additionally, nuclear lamin organization (**b**) and nuclear blebs (**c**) were quantified by counting the cells per field of the 40× microscopic view. Figures are representative of fully (**e**) or partially (**g**) organized nuclear lamin. Scale bar: 10 μm. An unpaired *t*-test was used. Data are shown as mean ± SEM (*n* = 80). **** *p* < 0.0001, ** *p* < 0.01.

Lamins are fundamental proteins that support nuclear stability, shape, structure, and pore positioning [12,16]. To test whether *LMNA*-KI affects nuclear morphometry, the organization of the nuclear lamina and the proportion of cardiomyocytes with nuclear blebbing were assessed (Figure 4a–c). Cardiomyocytes with *LMNA*-KI mutation had a significantly higher proportion of disorganized lamin distribution (WT 8.16%, *LMNA*-KI 17.71%, *p* < 0.0001) (Figure 4a,b) as evidenced by measuring the intensity of lamin through the nucleus (Figure 4d–g). Similarly, the percentage of nuclear blebbing was also significantly higher in cardiomyocytes derived from *LMNA*-KI mutated cells (WT 2.01%, *LMNA*-KI 11.34%, *p* < 0.0001) (Figure 4a,c).

### 4.5. LMNA-KI Cardiomyocytes Show Increased Functional Dysregulation under Isoprenaline Stress That Can Be Prevented by mTOR Inhibition

**Figure 5:**
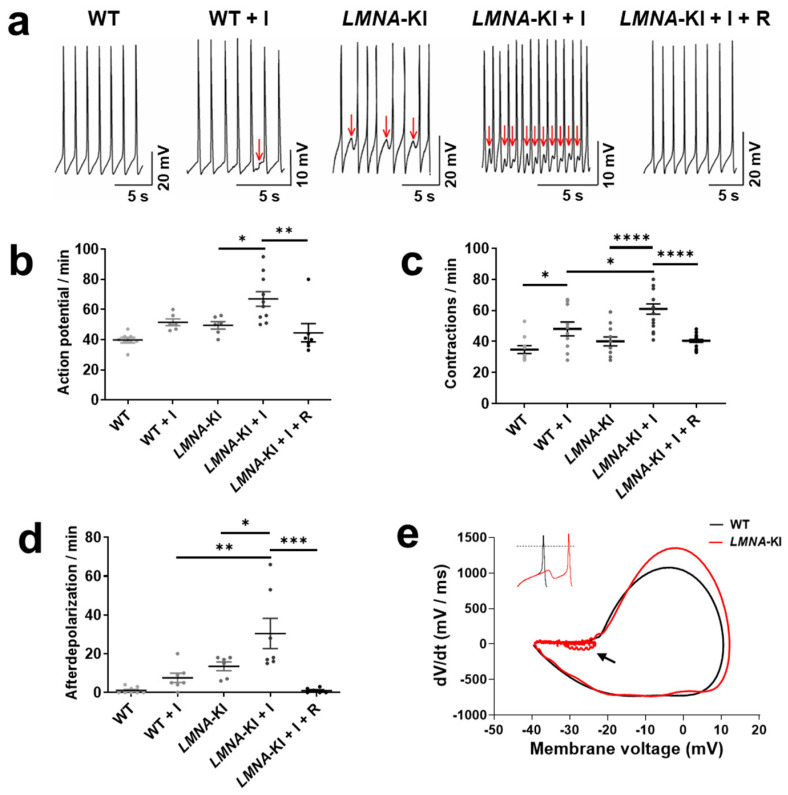
Functional characterization of cardiomyocytes differentiated from WT and *LMNA*-KI mES cells. (**a**) Electrophysiological recordings of spontaneous APs of WT.CMs, WT.CMs pre-incubated with isoprenaline, *LMNA*-KI.CMs, and *LMNA*-KI.CMs pre-incubated with either isoprenaline alone or with isoprenaline and rapamycin; red arrows indicate delayed afterdepolarization-like arrhythmias. (**b**–**d**) Frequency of APs, contractions, and delayed afterdepolarizations in WT.CM and *LMNA*-KI.CM under different conditions. (**e**) WT.CM (black line) and *LMNA*-KI.CM (red line) phase plot (dV/dt versus V) of a single AP; inset shows the respective AP traces (dotted line indicates 0 mV). Arrow indicates the delayed afterdepolarization-like arrhythmias in *LMNA*-KI.CM. One-way ANOVA and Tukey’s post hoc analysis were used. Data are shown as mean ± SEM (*n* = 6–19). **** *p* < 0.0001, *** *p* < 0.001, ** *p* < 0.01, * *p* < 0.05. I = isoprenaline, R = rapamycin, dV/dt = rate of action potential.

To characterize the effects of an mTOR inhibitor on the functional activity of WT.CMs and *LMNA*-KI.CMs, we examined the electrophysiological properties of single cells (Figure 5). Spontaneous action potential (AP) activity was recorded from cardiomyocytes without treatment and after 24 h of incubation with either β-adrenoceptor agonist isoprenaline alone or isoprenaline plus rapamycin (Figure 5a). Isogenic WT.CMs displayed regular APs with a firing frequency of 39.7 ± 1.6 APs/min, and contractions with a frequency of 34.8 ± 2.5 min^−1^. In comparison, *LMNA*-KI.CMs exhibited an increased frequency of APs (49.5 ± 2.4 APs/min) and of contractions (40 ± 2.9 min^−1^) (Figure 5b,c, Supplementary Videos S1–S5). Additionally, *LMNA*-KI.CMs also showed delayed afterdepolarization-like arrhythmias that appeared with a frequency of 13.5 ± 2.2 min^−1^ (Figure 5d). These afterdepolarization-like arrhythmias are illustrated in a phase plot in Figure 5e. The variability of individual APs of WT.CMs and *LMNA*-KI.CMs are illustrated in Supplementary Figure S2. The electrophysiological characteristics of *LMNA*-KI.CMs are consistent with a previous report of other *LMNA* mutants [17]. After 24 h of incubation with isoprenaline, WT.CMs displayed an increase in the frequency of APs (51.5 ± 2.2 APs/min) and of contractions (48.1 ± 4.4 min^−1^, *p* < 0.05) and delayed afterdepolarizations appeared at a frequency of 7.5 ± 2.4 min^−1^ (Figure 5b–d). Stimulation of *LMNA*-KI.CMs with isoprenaline even further increased all the analysed parameters compared with WT.CMs (67 ± 4.9 APs/min; 60.9 ± 3.2 contractions/min, *p* < 0.05; 30.4 ± 7.8 afterdepolarizations/min, *p* < 0.01) (Figure 5b–d). Importantly, the higher frequency of APs and contractions of *LMNA*-KI.CMs treated with isoprenaline were completely prevented by rapamycin co-treatment (44.5 ± 6.0 APs/min, *p* < 0.01; 40.4 ± 0.9 contractions/min, *p* < 0.0001), such that the values were comparable to WT.CMs without isoprenaline stimulation (*p* < 0.9) (Figure 5b,c). Strikingly, after rapamycin treatment, we also observed almost no afterdepolarization-like arrhythmias (1.0 ± 0.5 min^−1^; Figure 5a,d). These results suggest that the mTOR signaling pathway could be a target to modify the pro-arrhythmic activity of *LMNA*-KI.CMs. 

### 4.6. Relating the In-Vitro Data to Human Tissue

**Figure 6:**
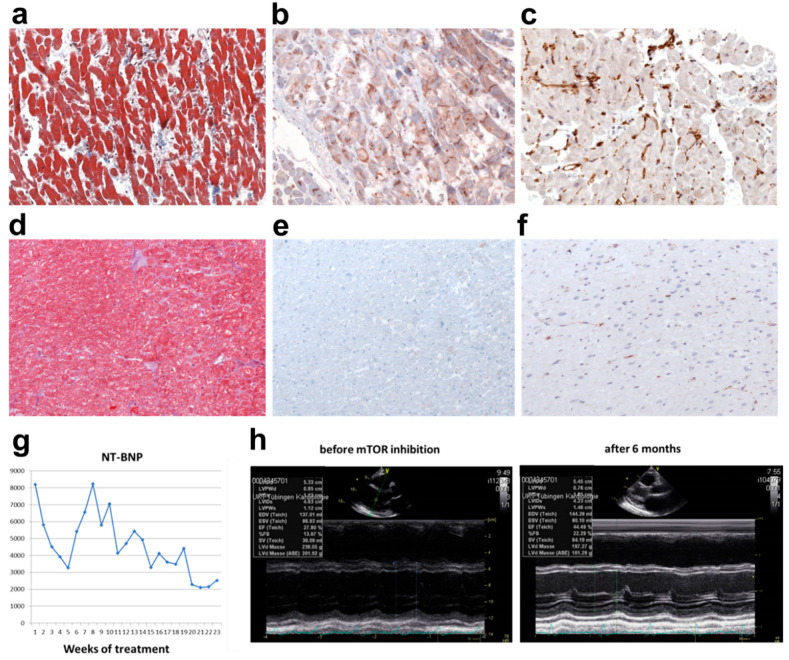
Patient biopsy data and treatment follow-up. Pathologic examination of patient biopsy from left ventricle was done with (**a**) Masson’s trichrome staining, (**b**) immunohistochemical staining using anti-phospho-mTOR Ab and (**c**) HLA-DR staining, illustrating mostly macrophages. Corresponding Masson’s trichrome (**d**), anti-phospho-mTOR (**e**) and HLA-DR (**f**) stainings from a healthy, 61-year-old male revealing normal stainings in the myocardium. The *LMNA*-affected DCM patient was treated off-label with the mTOR-inhibitor rapamycin at a serum concentration of 8 ng/mL for 6 months as an off-label treatment. (**g**) NT-BNP serum concentration dropped from 8120 ng/L to 2210 ng/L. Measurements of NT-BNP levels at an interval of 1 week are shown. (**h**) Echocardiographic information before and 6 months after the rapamycin treatment. NT-BNP = N-terminal brain natriuretic peptide.

On the evidence of mTOR activation in our in vitro model, we obtained cardiac biopsies from the patient. Masson’s trichrome staining revealed interstitial fibrosis (Figure 6a). Immunohistochemical stainings using an anti-phospho-mTOR antibody showed clear mTOR activation (Figure 6b), and macrophage infiltration as detected using an anti-HLA-DR antibody (Figure 6c) in the cardiac samples. Figure 6d–f show corresponding Masson’s trichrome, anti-phopho-mTOR and HLA-DR stainings from a healthy, 61-year-old male.

### 4.7. Gradual Recovery of the Patient with Lamin-Based DCM by Off-Label mTOR-Inhibition

Since all conventional treatment regimens were exhausted (the patient was not suitable for heart transplantation or left ventricular assist device implantation due to his pre-existing comorbidities) and the patient remained in severe clinical heart failure, an interdisciplinary team consisting of cardiologists, cardiac surgeons, geneticists, and anesthesiologists discussed the case in a “personalized medicine grand-round”. It was decided that based on the findings, that mTOR inhibition with rapamycin could be attempted in this case. 

Following informed, written consent, oral rapamycin at the serum concentration of 8 ng/mL was administered for a total of 6 months. After 6 months, the patient’s symptoms had improved to NYHA class II and he was able to return to work. This was accompanied by a drop in the serum NT-pro-BNP concentration (from 8120 ng/L to 2210 ng/L), and an improvement in LVEF from 28% to 45% (Figure 6g,h). The patient was discharged from the hospital and was able to resume his work. Relevant side effects of rapamycin were not observed.

## 5. Discussion

**Figure 7:**
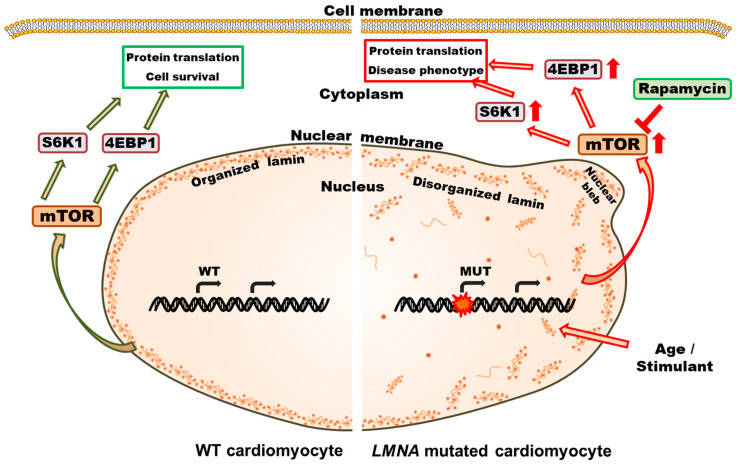
Schematic diagram of a putative signaling pathway in cardiomyocytes with *LMNA* mutation. Factors like age or stimulants affect the gene expression of *LMNA* mutant cells. Dysregulation of gene expression from mutant alleles causes higher activation of the mTOR signaling pathway. This dysregulation causes protein translation imbalance, lamin disorganization, and nuclear blebbing and adds to the disease phenotype. mTOR-inhibitor rapamycin is effective in checking the cellular effects of *LMNA* mutation.

Our work has shown four important observations. We found that cardiomyocytes differentiated from *LMNA*-KI mES cells containing the patient-specific LMNA E161K mutation showed increased mTOR phosphorylation when stimulated with isoprenaline. We were also able to reveal a higher percentage of nuclear disorganization and nuclear blebbing in *LMNA*-KI cardiomyocytes. Finally, we showed that mTOR inhibition prevented functional dysregulation in vitro (Figure 7), and that, using this information, mTOR inhibition led to a remarkable improvement in clinical status in a patient with the LMNA E161K mutation and severe heart failure. As such our data uniquely describe the complete process from genetic analysis through to bedside application of the findings.

### 5.1. Dilated Cardiomyopathy (DCM)

Although DCM is a clinical syndrome with a characteristic phenotype, it can be the result of multiple underlying pathophysiologies most of which are poorly understood. Around 6% of cases of the familial form of DCM are the consequence of mutations in the nuclear membrane protein Lamin A/C [11]. Patients with *LMNA* mutations frequently suffer a fulminant heart failure syndrome along with multiple organ dysfunction [7]. There are no approved personalized approaches to the management of DCM, rather standard therapy follows the ‘four pillars’ approach recommended by the European Society of Cardiology for HFrEF (ESC HF guidelines 2021). 

### 5.2. Generating the Specific Mutation in Wild-Type Cells

We exploited the CRISPR-Cas9 gene editing technology to generate the patient *LMNA*-KI mutation in WT mES cells. CRISPR-Cas9 is increasingly gaining popularity to create experimental models to study pathogenicity and cellular changes arising from gene mutations [18]. WT and *LMNA*-KI mutated mES cells were further differentiated into cardiomyocytes using the previously described hanging drop method [14]. This protocol is a standard method of cardiomyocyte differentiation via embryoid body formation [19,20]. Cardiomyocytes derived from both WT and *LMNA*-KI mutated mES cells were examined for markers of several cell types ranging from early embryonic ectoderm to cardiomyocytes. Significant upregulation of *Oct3/4* and *Nanog*, both markers for early embryonic ectoderm [21,22], in undifferentiated cells, show the degree and specificity of mES cells. On the other hand, markers for cardiomyocyte differentiation, *Gata4*, *Tbx20*, *cTNT*, *α-Myh6*, *Mlc2a,* and *Mlc2V*, were significantly upregulated in differentiated WT and *LMNA*-KI cells. Tbx20, which is expressed within E7.5, has been known to interact with several other proteins such as Nkx2-5, Gata4, and Gata5 and regulates the expression of various cardiac genes as well as coordinates cell proliferation and the process of cardiac differentiation [23]. Also, in mature cardiomyocytes, the expression of *cTNT* and *α-Myh6* is crucial to maintaining the normal heart function [21,24]. Atrial and ventricular subtypes of cardiomyocytes can be distinguished by the expression of either *Mlc2a* (for atrial cardiomyocytes) or *Mlc2V* (for ventricular cardiomyocytes). However, these markers are not completely specific, and can be present in both lineages [25]. Upregulation of cardiomyocyte-specific gene markers, thus, shows successful cardiomyocyte differentiation from WT or *LMNA*-KI mutated mES cells.

### 5.3. The Morphological Effects of LMNA-K1

Since lamins are important proteins to support the shape, size, and stability of the nucleus [12,17], we examined if the heterozygous allele impacts the organization of the nucleus. Interestingly, cardiomyocytes derived from *LMNA*-KI mES cells showed a higher proportion of disorganized lamin distribution as well as a higher percentage of nuclear blebbing. Morphological changes in abnormal forms such as nuclear blebbing, herniations, honeycomb/donut-like structures or any combinations of these categories in the nucleus due to mutations in the *LMNA* gene are well described. Smaller volumes of chromosome 13 and abnormal nuclear shapes have been described in LMNA E161K mutant fibroblast [13]. However, these abnormalities are dependent on the type of cells, and location of the mutation and are not observed in all laminopathic cells [26]. These dysmorphic nuclei are also reported in fibroblasts or lymphoblastoid cells obtained from an *LMNA* mutated patient [27] indicating their clinical significance.

### 5.4. The Electrophysiological Effects of LMNA-K1

Electrophysiologically, the *LMNA*-KI mutation generates an increased pro-arrhythmic activity in the cardiomyocytes derived from mES cells, characterized by an increased AP frequency, delayed afterdepolarizations, and an increase in the contraction frequency; this arrhythmic phenotype is similar to another *LMNA* mutation [17]. The pro-arrhythmic activity of *LMNA*-KI.CMs became severe after 24 h of treatment with the non-selective β-adrenoceptor agonist isoprenaline and with higher activation of the mTOR signaling pathway. Interestingly, we observed that with rapamycin treatment, we were able to completely recover the electrical activity of *LMNA*-KI.CMs to that of WT.CMs. Rapamycin, in this context, allowed us to explore the possibility to use a specific inhibitor of the mTOR signaling pathway as a pharmacological treatment for *LMNA*-related cardiomyopathy.

Cardiomyocytes generated from mES cells were further subjected to the non-selective β-adrenoceptor agonist isoprenaline. We have previously used isoprenaline as a factor to initiate DCM [1,28]. Cardiomyocytes derived from *LMNA*-KI mES cells showed increased phosphorylation of mTOR complex-1 (mTORC1) and 4EBP1 whereas, phosphorylation of mTOR complex-2 remained unaffected (Supplementary Figure S3). Thus, this study confirms an increased degree of the mTORC1 signaling pathway activation in the LMNA E161K mutation. We, therefore, opted for rapamycin to inhibit this signaling pathway. Our results showed a clear and strong inhibition of mTORC1, P70S6K, and 4EBP1 phosphorylation with rapamycin application in cardiomyocytes stimulated with isoprenaline. Preclinical studies in *LMNA* knock-out mice have shown the efficacy of rapamycin in preventing elevated mTORC1 signaling, thereby providing a potential remedy for the cure of DCM, skeletal muscle dystrophy, and lipodystrophy, and significantly increasing the survival time [29].

### 5.5. Clinical Application

With laboratory evidence of increased mTOR activity and reversal of cellular functions by mTOR inhibitor, and supported by the hospital ethics board, we administered the mTOR inhibitor rapamycin, in a compassionate and off-label application to the *LMNA*-KI patient. This was followed by a steady improvement in clinical status and cardiac function on echocardiography.

We acknowledge that our work has limitations. First, although electrophysiological measurements revealed the typical character of cardiomyocytes, we cannot exclude that these cells have not or cannot become fully mature adults CMs under the current defined condition. Second, differentiation happened randomly with varying percentages of atrial or ventricular lineages. Third, an in vitro model cannot recapitulate the actual disease phenotype in an organism. Further, increased expression of *microtubule-associated protein-1 light chain 3B* (*LC3B*) (Supplementary Figure S4) and a study done by Choi et al. [30] suggest that modulation of defective autophagy might be an interesting target for pharmacological interventions in DCM arising from *LMNA* mutations. However, further research on this pathway was beyond the scope of this study. 

The patient was under optimal medical treatment at the time of our study. Treatment guidelines for heart failure have changed and additional classes of agents have become the standard of care since our study. It is unclear, if mTOR inhibition would have had an additional benefit in the four-pillar model now proposed by the ESC. 

Finally, the success of rapamycin treatment in one patient with a specific *LMNA* mutation may not be transferable to other *LMNA* mutations. Moreover, since treatment was terminated 6 months after initiation of rapamycin according to our ethics protocol, any conclusion on long-term effects cannot be made by our study. This requires further pre-clinical and clinical evaluation.

## 6. Conclusions

We successfully created and characterized a patient-specific *LMNA*-KI in vitro model that provided a comprehensive basis to understand cellular changes arising from an *LMNA*-KI mutation in a patient with severe and treatment-resistant familial DCM, and then allowed us to provide a well-tolerated, focused, and ultimately successful therapeutic approach. Our study describes how the future of personalized care for selected patients with HFrEF could look, with targeted therapies based upon genetic profiling and a comprehensive understanding of the molecular basis of their disease. 

## 7. Perspectives

### 7.1. Clinical Competencies

This study describes a personalized medicine approach for a DCM patient with *LMNA*-related cardiomyopathy using mES cell-based disease modeling. This approach considers the genetic basis of the disease where clinical effects are often heterogeneous. The protocol may serve as a template for other patients with familial heart failure and stresses the need for consequent genetic screening in patients with unclear cardiomyopathy.

### 7.2. Translational Outlook

Increased mTOR signaling is evident in a DCM patient with an LMNA E161K mutation as well as in cardiomyocytes derived from mES cells with the patient-specific *LMNA* mutation. LMNA E161K mutation results in impaired electrophysiological properties and disorganisation of nuclear lamin in vitro. The mTOR-inhibitor rapamycin was able to rescue the phenotype in vitro and also improved the clinical conditions of a DCM patient with an LMNA E161K mutation. Further pre-clinical, as well as clinical studies, are required to establish a precision treatment.

## Data Availability

Not applicable.

## Supplementary Materials

The following are available online at https://www.mdpi.com/article/10.3390/jpm12071149/s1, Table S1: List of primers used for expression analysis, Figure S1: Expression of markers for differentiated and undifferentiated WT and *LMNA*-KI mES cells, Figure S2: Action potential distribution plot of WT.CM and *LMNA*-KI.CM, Figure S3: Western blot images of protein expression in cardiomyocytes with and without isoprenaline (I) stimulation, Figure S4: Expression of autophagy markers in *LMNA*-KI cardiomyocytes, Supplementary Methods S1: Descriptive methodology used for electroporation, real-time PCR, Western blot, and immunohistochemistry, Supplementary Result S1: Validation of cardiomyocytes differentiated from WT and *LMNA*-KI mES cells, Video S1: The contractions of cardiomyocytes differentiated from wild type mouse embryonic stem cells, Video S2: The contractions of cardiomyocytes differentiated from wild type mouse embryonic stem cells and stimulated with isoprenaline (2 μM final concentration) for 24 h, Video S3: The contractions of cardiomyocytes differentiated from LMNA E161K mouse embryonic stem cells, Video S4: The contractions of cardiomyocytes differentiated from LMNA E161K mouse embryonic stem cells and stimulated with isoprenaline (2 μM final concentration) for 24 h, Video S5: The contractions of cardiomyocytes differentiated from LMNA E161K mouse embryonic stem cells and co-treated with isoprenaline (2 μM final concentration) and rapamycin (500 nM final concentration) for 24 h.

## Abbreviations

DCM	dilated cardiomyopathy
LMNA	lamin A/C
mES cell	mouse embryonic stem cell
KI	knock in
CRISPR	clustered regularly interspaced short palindromic repeats
mTOR	mammalian target of rapamycin
MEF	mouse embryonic fibroblast
sgRNA	single guide RNA
ATCC	American type culture collection
ACE	angiotensin converting enzyme
CRT-D	cardiac resynchronisation therapy—implantable cardioverter defibrillator
LVEDD	left ventricular end diastolic dimension
AP	action potential
CM	cardiomyocyte
