# Supplementary material for: Personalized Medicine Approach in a DCM Patient with LMNA Mutation Reveals Dysregulation of mTOR Signaling
Source: J Pers Med. 2022 Jul 15;12(7):1149. doi: 10.3390/jpm12071149 (PMC9323361; doi:10.3390/jpm12071149)
Supplement: Supplementary file 1 [file jpm-12-01149-s001.zip › jpm-1806438-supplementary.pdf]

Supplementary table

Table S1. List of primers used for expression analysis.

S. No.	Name of the protein	Name of the gene	Forward primer (5' - 3')	Reverse primer (5' - 3')
1	Oct3/4	*Oct3/4*	TGTGGACCTCAGGTTGGACT	TTTCATGTCCTGGGACTCCTC
2	Nanog	*Nanog*	CCTCGCCATCACACTGACAT	GGCGAGGAGAGGCAGC
3	Gata4	*Gata4*	TGGCCAGGACTGCCG	TCACCCACCGGCTAAAGAAG
4	Tbx20	*Tbx20*	ACGGCCTCCTTGCTCAATC	ACAAGACCTCATTCCCTCTCAA
5	cTNT	*Tnnt2*	GACAGGATCGAAAAGCGTCG	ACCCTCCAAAGTGCATCATGT
6	αMyh6	*Myh6*	AAGGTGAAGGCCTACAAGCG	GTCGTGCATCTTCTTGGCAC
7	Mlc2a	*Myl7*	GGTCCCATCAACTTCACCGT	GTTGCTCTACCTCAGCAGGA
8	Mlc2v	*Myl2*	CTCCAAAGAGGAGATCGACCA G	TGTTTATTTGCGCACAGCCC
9	Lamin A/C	*LMNA*	AGAGGGAATAGGAGGCAAAG	AGAGCAGAGTTGCAGAGAG
10	P62	*P62*	AGAATGTGGGGGAGAG TGTG	TTTCTGGGGTAGTGGG TGTC
11	LC3A	*LC3A*	CTGTAAGGAGGTGCAG CAGA	CCGGATGATCTTGACC AACT
12	LC3B	*LC3B*	AGATAATCAGACGGCG CTTG	ATTGCTGTCCCGAATG TCTC

Supplementary table S1: Primers used for real-time PCR on murine cDNA (1-8), surveyor nuclease assay (9) and for expression of autophagy markers (10-12). Oct3/4: Octamer-binding transcription factor 3/4; Nanog: Nanog Homeobox; Gata4: GATA binding protein 4; Tbx20: T-Box transcription factor 20; cTnT: Cardiac muscle isoform of troponin T; TNNT2: Troponin T2, cardiac type; αMyh6: Myosin heavy chain, α isoform; Mlc2a: Myosin light chain 2, atrial isoform; Myl7: Myosin light chain 7; Mlc2v: Myosin light chain 2, ventricular isoform Myl2: Myosin regulatory light chain 2, ventricular/cardiac muscle isoform; p62: ubiquitin-binding protein p62; LC3A: Microtubule-associated protein 1A/1B-light chain 3A; LC3B: Microtubule-associated protein 1A/1B-light chain 3B. The primer sequence for p62, LC3A and LC3B has been cited from Choi et al (Choi et al, 2012)

Supplementary methods S1: Descriptive methodology used for electroporation, real-time PCR, Western blot, and immunohistochemistry.

Electroporation

NIH/3T3 or WT mES cells grown on 6 well plates were washed twice with 1x PBS before electroporation. 5µl of 100µM tracr-RNA (IDT-DNA) and 5µl of 100µM crRNA (IDT-DNA) were mixed with 10µl IDT duplex buffer and incubated for 20 minutes in room temperature. 5µl Cas9 nuclease (IDT-DNA) and 10µl IDT duplex buffer was added and incubated for 30 minutes in room temperature. This was mixed with 82µl nucleofection buffer (P3 primary cell 4D-nucleofector™ kit, Lonza, Switzerland), 18µl supplement and 3µl of 100µM donor DNA (see supplement for the donor DNA sequence). The donor DNA contains the patient specific *LMNA* mutation together with two additional silent mutation for deactivating the protospacer adjacent motif region and for the facilitation of clone selection via restriction enzyme digestion. This solution was then mixed with cell pellet ($1x10^6$ cells) and electroporated via Amaxa™ 4D-Nucleofector™ (Lonza) using the manufacturer's guide. The electroporated cells were then incubated at room temperature for 15 minutes. NIH/3T3 cells were seeded in T25 while WT mES cells were seeded into 10 cm petri dishes at the rate of 10,000 cells per petri dish.

Real-time PCR

RNA isolation was done by using Rneasy mini kit (Qiagen 74106). The pellets from mES cells and differentiated cardiomyocytes from mES cell were homogenized by using QIA shredder (Qiagen 74106). Other steps for isolation were done according to the protocol. The RNA samples were then treated with Dnase I (Sigma Aldrich AMPD1) to digest the genomic DNA. Iscript cDNA synthesis kit (Biorad 1708891) was used to prepare cDNA. qPCR was performed in viiA7 real-time PCR system (Applied Biosystem). All primers used for qPCR are listed in the supplement.

Western blot

The cardiomyocytes differentiated from WT and *LMNA* mutant mES cells were used for the extraction of total protein. Protein concentration was determined by bicinchoninic acid (BCA)

assay. 10μg of protein was used to load the samples for gel electrophoresis. Blotting of separated protein was done on nitrocellulose membrane. Blots were incubated with primary or secondary antibodies and protein expressions were detected in Bio-Rad western blot imager.

The cardiomyocytes differentiated from WT and *LMNA* mutant mES cells were washed with Ca^{2+}-free phosphate buffered saline. Cell lysis was done with the reagent prepared by mixing Pierce RIPA buffer (Thermofisher, 89900), PhosSTOP™ (Sigma Aldrich, 4906837001) and cOmplete™ Protease Inhibitor cocktail (Sigma Aldrich, 11697498001). The sonicated mixture was then centrifuged at 14800rpm at 4°C for 10 minutes. The protein concentration of the supernatant collected was determined by bicinchoninic acid (BCA) assay. The loading protein sample was prepared by mixing 10μg of protein and 4x sample loading dye. The gel electrophoresis was performed in a gel cassette with 5% stacking gel and 10% separating gel with 80 volts through stacking gel and 100 volts through separating gel. Blotting of the separated protein was done on nitrocellulose membrane. Blocking was done with 5% BSA. All the antibodies were diluted in 2% BSA and blots were incubated with primary antibodies at room temperature for 1 hour. The primary antibodies used were: recombinant anti-mTOR antibody (Y391) (ab32028, Abcam, 1:2000), phospho-mTOR (Ser2448) antibody (#2971S, Cell signaling, 1:1000), phosphor-mTOR (Ser2481) antibody (#2974, Cell signaling, 1:1000), 4E-BP1 antibody (#9452S, Cell signaling, 1:1000), phospho-4E-BP1 (Ser65) antibody (#9451S, Cell signaling, 1:1000), p70(S6K) polyclonal antibody (14485-1-AP, Proteintech, 1:5000), phospho-p70 S6 kinase (Thr 389) antibody (#9205S, Cell signalling, 1:1000), beta actin monoclonal antibody (66009-1-Ig, Proteintech, 1:8000). Blots were washed with TBST (1X Tris-buffered saline with 0.1% Tween 20 detergent) buffer between each incubation step. Blots were incubated with secondary antibodies for 1 hour. The secondary antibodies used were: anti-rabbit IgG, HRP-linked antibody (#7074S, Cell signalling, 1:10000) and anti-mouse IgG, HRP-linked antibody (#7076S, Cell signalling, 1:10000). Protein expressions were detected in Bio-Rad western

blot imager using SuperSignal^{TM} West Pico PLUS chemiluminescent substrate (Thermofisher).

Immunohistochemistry

The cardiomyocytes differentiated from WT and *LMNA* mutant mES cells were seeded in 12 well plate containing sterile coverslips. After 12 days of incubation, cells were fixed with 4% formaldehyde for 20 minutes. After washing the cells with PBS, permeabilization was done with 0.1% Triton X-100 (prepared in PBS) for 10 minutes. Again, after washing with PBS, cells were blocked with 5% BSA (prepared in PBS) for 30 minutes. Overnight incubation was done with primary antibodies, either actinin (anti-sarcomeric alpha actinin antibody, EA-53, ab9465, Abcam, 1:200) or Lamin A/C (E1) antibody (sc-376248, Santa Cruz, 1:200) at 4°C. After washing with PBS, cells were incubated with FITC-conjugated AffiniPure donkey anti-mouse IgG (715-095-150, Jackson Immunoresearch, 1:400) or Alexa Fluor 594 (A11005, Invitrogen, 1:400). Counterstaining of nucleus was done with 4'6-diamidino-2-phenylindole (DAPI) (Invitrogen, D1306). The coverslips were fixed in the slide using Vectashield Antifade mounting medium (Vector laboratories, H-1000). Cells were imaged using Leica STELLARIS 8 DIVE FALCON two-photon microscope, using Spectra-Physics X3 tuneable ultrafast laser, HC IRAPO L 25x/1.00W motCORR objective and 4Tune HyD RLD detectors, adjusted for DAPI (393-503nm) and AF594 (588-664nm) detection. 800nm excitation wavelength was used.

The heart biopsy tissues from the DCM patient or healthy control were fixed in 4% formaldehyde and embedded in paraffin. All stainings were performed in 4µm deparaffinised tissue sections. Masson's trichrome staining was done following standard staining protocol. For MHC (major histocompatibility complex) class II and phospho-mTOR stainings, immunohistochemical analysis was performed on an automated immunostainer following the manufacturer's protocol (Benchmark, Ventana medical Systems, Tucson, AZ) and using the ultraView detection system and diaminobenzidine as substrate. The primary antibody used were mouse anti-human HLA-DR (Human Leukocyte Antigen - DR isotype) Ab (clone

TAL 1B5, 1:50, Dako) and anti-phospho-mTOR (Ser2448) (49F9) rabbit mAB (#2976, Cell Signaling, Danvers, MA, 1:100). Secondary antibodies used were against anti-mouse and anti-rabbit (Sigma Aldrich, A3682, A0545). Slides were counterstained with hematoxylin and images were taken by using a Zeiss microscope.

Supplementary result

Supplementary result S1: Validation of cardiomyocytes differentiated from WT and *LMNA*-KI mES cells

Wild type and *LMNA*-KI cardiomyocytes (WT.CM and *LMNA*-KI.CM) were analysed for differentiation markers after 12 days of differentiation process. Expression of markers for undifferentiated cells *Oct3/4* (33.3 fold in WT.CM, and 328.2 fold in *LMNA*-KI.CM, $p<0.0001$) and *Nanog* (39.0 fold in WT.CM and 279.3 fold in *LMNA*-KI.CM, $p<0.0001$) were significantly decreased in cardiomyocytes derived from either WT cells or *LMNA*-KI mES cells (Supplement fig. 1a). Furthermore, the expressions of markers specific to cardiac mesoderm/progenitors *Gata4* (7.9 fold in WT.CM and 19.4 fold in *LMNA*-KI.CM, $p<0.0001$) and *Tbx20* (8.6 fold in WT.CM, $p<0.001$ and 10.9 fold in *LMNA*-KI.CM, $p<0.01$) were significantly increased in WT.CM and *LMNA*-KI.CM (Supplement fig. 1b). Cardiomyocytes specific markers *cTNT* (46.4 fold in WT.CM and 82.1 fold in *LMNA*-KI.CM, $p<0.0001$) and *αMyh6* (10.6 fold in WT.CM, $p<0.01$ and 63.4 fold in *LMNA*-KI.CM, $p<0.0001$) as well as markers specific to cardiomyocytes atrium *Mlc2a* (1.1fold in WT.CM, $p<0.01$ and 3.8 fold in *LMNA*-KI.CM, $p<0.0001$) or ventricles *Mlc2V* (71.1 fold in WT.CM and 120.3 fold in *LMNA*-KI.CM, $p<0.01$) were significantly increased in the differentiated cells (Supplement fig. 1c-d).

Supplementary figures

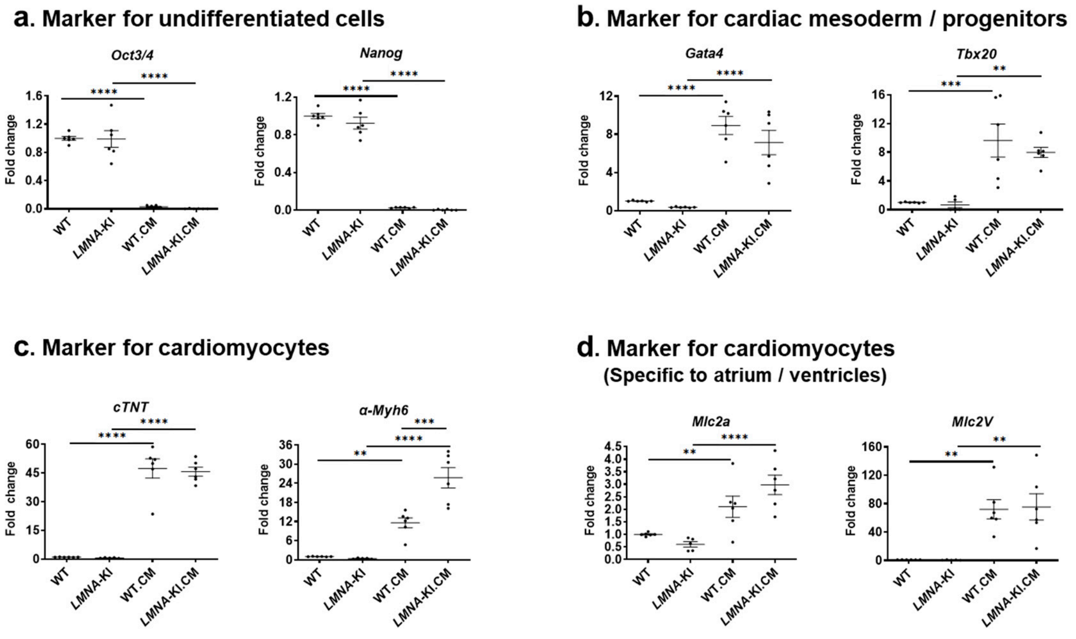

Figure S1: Expression of markers for differentiated and undifferentiated WT and *LMNA*-KI mES cells. WT and *LMNA*-KI mES cells were differentiated into cardiomyocytes and mRNA levels were measured for different markers on day 12. The markers measured were specific for (a) undifferentiated cells (*Oct3/4* and *Nanog*), (b) cardiac mesoderm / progenitors (*Gata4* and *Tbx20*), (c) cardiomyocytes (*cTNT* and *α-Myh6*), and (d) atrium or ventricles (*Mlc2a* and *Mlc2V*). One way ANOVA and Tukey's post hoc analysis was used. Data are shown as mean ± SEM (n = 6). ****p<0.0001, ***p<0.001, **p<0.01.

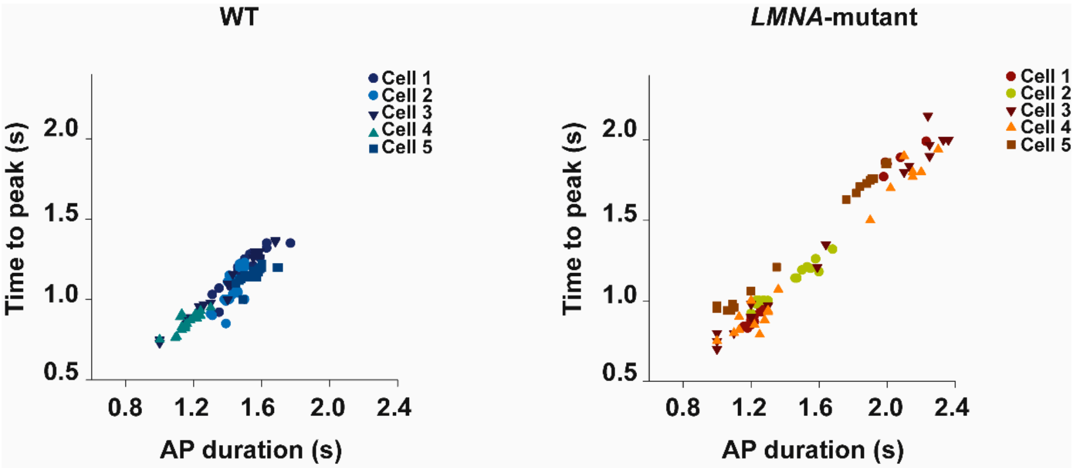

Figure S2. Action potential distribution plot of WT.CM and *LMNA*-KI.CM. Variability of duration vs time to peak of individual action potentials recordings from single

cardiomyocytes during 30s (5 cells per condition). Values of WT.CMs show a more narrow distribution compared to *LMNA*-KI.CMs.

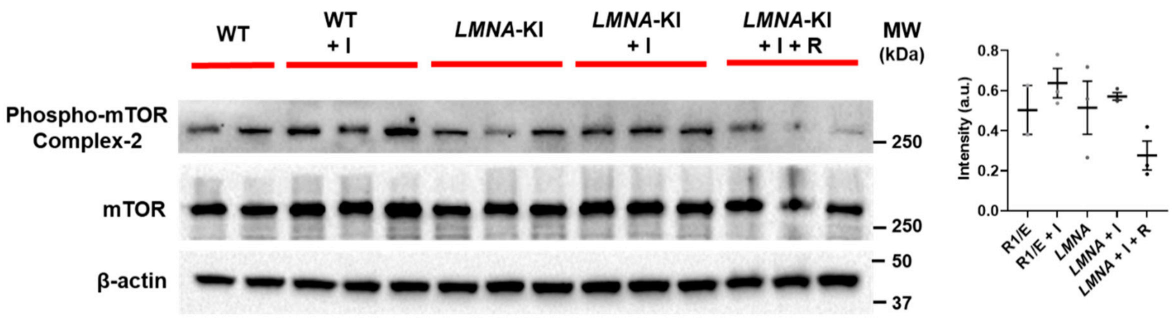

Figure S3. Western blot images of protein expression in cardiomyocytes with and without isoprenaline (I) stimulation. WT and *LMNA*-KI mES cells were differentiated into cardiomyocytes and stimulated on day 12 with either isoprenaline (I) or isoprenaline together with rapamycin (R). Western blot images of total protein levels of phosphor-mTOR complex 2 or mTOR. β-actin is used as a loading control. Right panels shows the densitometric quantification of phosphorylated mTOR complex-2, measured by ImageJ program. One way ANOVA and Tukey's *post hoc* analysis was used. Data are shown mean ± SEM (n = 2-3).

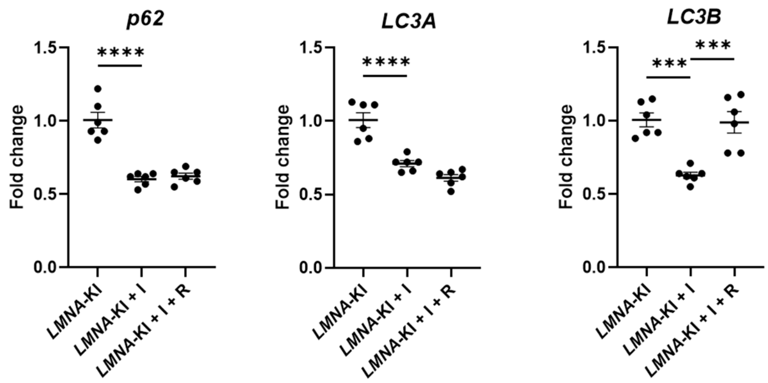

Figure S4. Expression of autophagy markers in *LMNA*-KI cardiomyocytes. *LMNA*-KI mES cells were differentiated into cardiomyocytes and stimulated on day 12 with either isoprenaline (I) or isoprenaline together with rapamycin (R). The markers measured were

specific for autophagy. One way ANOVA and Tukey's post hoc analysis was used. Data are shown as mean±SEM (n=6). ****p<0.0001, ***p<0.00.

Single guide RNA2 (sgRNA2): Alt-R® CRISPR-Cas9 crRNA (IDT-DNA)

Sequence (sgRNA2):

/AlTR1/rCrArGrUrGrArGrArArGrCrGrCrArCrArUrUrGrGrGrUrUrUrArGrArGrCrUrArUrGrCr
U/AlTR2/

Donor DNA sequences (IDT-DNA)**:**

Wild type:

TCCAAGGAAGCTGCCCTGAGCACTGCTCTCAGTGAGAAGCGCACATTGGAGGGCGAG
CTCCATGACCTGCGGGGGCAGGTAGCCAAGGTAGGCCGCTGTCCTGTGACCCCAGTG
A

Top donor (mutations are marked with small alphabets):

TCACTGGGGTCACAGGACAGCGGCCTACCTTGGCTACCTGCCCCCGCAGGTCATGaA
GCTtGCCtTCCAATGTGCGCTTCTCACTGAGAGCAGTGCTCAGGGCAGCTTCCTTGGA

Bottom donor (mutations are marked with small alphabets):

TCCAAGGAAGCTGCCCTGAGCACTGCTCTCAGTGAGAAGCGCACATTGGAaGGCaAGC
TtCATGACCTGCGGGGGCAGGTAGCCAAGGTAGGCCGCTGTCCTGTGACCCCAGTGA

Videos: Videos 1-5, attached as separate *.mp4 files.

Video legends

Video S1: The video represents the contractions of cardiomyocytes differentiated from wild type mouse embryonic stem cells.

Video S2: This video represents the contractions of cardiomyocytes differentiated from wild type mouse embryonic stem cells and stimulated with isoprenaline (2μM final concentration) for 24 hours.

Video S3: This video represents the contractions of cardiomyocytes differentiated from LMNA E161K mouse embryonic stem cells.

Video S4: This video represents the contractions of cardiomyocytes differentiated from LMNA E161K mouse embryonic stem cells and stimulated with isoprenaline (2μM final concentration) for 24 hours.

Video S5: This video represents the contractions of cardiomyocytes differentiated from LMNA E161K mouse embryonic stem cells and co-treated with isoprenaline (2μM final concentration) and rapamycin (500nM final concentration) for 24 hours.
